# Fast-Dissociating High-Supply Peptides as Contributors to MHC-I Antigen Presentation

**DOI:** 10.3390/cells15191735

**Published:** 2026-09-23

**Authors:** Om H. Gandhi, Michael E. Bryan, Malcolm J. W. Sim, Tim Elliott

**Affiliations:** Centre for Immuno-Oncology, University of Oxford, Oxford OX3 7DQ, UK; om.gandhi@univ.ox.ac.uk (O.H.G.); michael.bryan@new.ox.ac.uk (M.E.B.); malcolm.sim@immonc.ox.ac.uk (M.J.W.S.)

**Keywords:** immunopeptidome, MHC class I, peptide supply, FDHS, defective ribosomal products, antigen presentation, neoantigen, peptide–MHC stability, mass spectrometry, tapasin, ribosome-associated quality control

## Abstract

**Highlights:**

**What are the main findings?**

- Peptide–MHC stability is often used to predict presentation of specific peptide–MHC class I complexes. We argue that peptide supply is also an essential component of antigen presentation and may allow surface display of fast-dissociating, high-supply (FDHS) peptides.
- Techniques to detect peptide–MHC-I complexes can lose short-lived ligands during sample preparation, so FDHS peptides may be under-represented in immunopeptidomes.

**What are the implications of the main findings?**

- Epitope prioritisation that ranks candidates on predicted binding affinity alone may discard FDHS candidates. Tumour-selective supply also raises the possibility of FDHS targets with limited thymic presentation and reduced central tolerance.
- We specify the measurements of peptide supply, dissociation rate and surface copy number, made in a single system, that would test the relevance of FDHS peptides to immunopeptidomes.

**Abstract:**

The major histocompatibility complex class I (MHC-I) immunopeptidome is dynamic and determined by numerous biochemical and cellular features. Computational tools to predict peptide–MHC-I binding focus on the affinity of a specific peptide–MHC interaction; separate tools predict complex stability or presentation. Methods used to define the immunopeptidome may preferentially recover stable complexes, and presentation predictors trained on those data may inherit that bias. However, peptides that dissociate rapidly but are generated at high rates could reach surface densities sufficient for CD8^+^ T cell recognition. Here, we examine evidence relevant to these fast-dissociating high-supply (FDHS) peptides and develop a two-axis model, whereby peptide supply is considered alongside stability. We examine how defective ribosomal products and ribosome-associated quality control supply peptides for MHC-I loading. We set out the limits of tapasin-mediated editing and compare the model with the binding, stability and presentation predictors now in use. Experiments have linked peptide abundance and complex stability to relative presentation, but the extent of compensation for endogenous FDHS peptides remains to be quantified. We present experimental systems to test the magnitude and limits of this compensation, specifying the key measurements. We also propose that FDHS ligands generated under tumour-specific translational stress could be less constrained by central tolerance if their presentation is insufficient during thymic selection. We believe this model has implications for understanding the broader immunopeptidome, how it contributes to innate and adaptive immunity, and designing better immunotherapies.

## 1. Introduction

Major histocompatibility complex class I (MHC-I) peptide loading is a competitive process where peptides with higher affinity for MHC-I are preferentially selected for cell-surface presentation [1,2,3,4,5]. Thus, peptides that bind MHC-I weakly can be excluded by affinity-based candidate filters [6], although weak binding does not establish low immunogenicity or rapid dissociation. However, cluster of differentiation 8-positive (CD8^+^) T cells can detect low ligand numbers, with as few as three peptide–MHC-I (pMHC-I) complexes at the contact interface sufficient for a differentiated cytotoxic T lymphocyte (CTL) to kill a target it is already engaging [7]. Additionally, whether a peptide is presented for long enough and at sufficient density for T cell recognition depends on the balance between the delivery of new complexes and loss of existing ones.

Delivery of peptides through the conventional pathway depends on substrate input to the proteasome, transport by the transporter associated with antigen processing (TAP), and loading in the endoplasmic reticulum (ER) [1,2,3]. In theory, where supply is high enough, a pMHC-I complex with a half-life of an hour or two could reach sufficient density for recognition by CD8^+^ T cells. However, such fast-dissociating high-supply (FDHS) peptides may be under-recorded because recovery by immunopeptidomics and some peptide-binding assays depends on complex survival during sample handling.

FDHS describes a proposed combination of rapid peptide dissociation and high peptide supply, without a universal cutoff for either quantity. Peptide supply refers to the rate at which copies of a particular peptide sequence enter antigen processing within proteins or other substrates. Only a fraction of those copies reaches the surface as pMHC-I. Hence, source-protein abundance alone does not establish peptide input or the fraction successfully presented. This relationship could also contribute to immunogenicity: increased supply in tumours or during infection could expose ligands that were poorly presented during thymic selection, leaving responsive T cells available in the mature repertoire.

There is evidence relevant to immunogenic FDHS peptides in the literature. Cells lacking the ER aminopeptidase associated with antigen processing (ERAAP), the mouse counterpart of human ER aminopeptidase 1 (ERAP1), display novel unedited ligands whose functional presentation declines within hours after new delivery is blocked. However, these cells elicit CD8^+^ and B cell responses potent enough to raise an antibody against unedited peptide–MHC (pMHC) complexes in MHC-matched animals [8,9]. Further, reducing ERAAP in the CT26 tumour model increased generation of the antigen GSW11, converting the tumour into one that induces protective CD8-dependent immunity [10]. CD8^+^ T cells specific for influenza A nucleoprotein NP366–374 dominated the secondary response despite a shorter surface-complex half-life than the polymerase acidic protein epitope PA224–233 in a separate loading assay [11,12,13]. During chronic lymphocytic choriomeningitis virus (LCMV) infection, T cells specific for the glycoprotein epitope GP276–286 become a dominant surviving population but remain functionally impaired [14]. These examples establish distinct effects of pMHC-I complex lifetime, antigen exposure and recognition; none alone demonstrates high supply compensating for rapid dissociation.

The limitations of current methods could contribute to under-recording of short-lived ligands. Sequencing of naturally presented ligands established MHC-binding motifs, and mass spectrometry (MS)-based immunopeptidomics subsequently enabled large datasets for binding and presentation prediction [15,16,17,18,19]. However, immunoprecipitation coupled to MS (IP-MS) cannot recover ligands that dissociate and are discarded during sample handling. The size of this loss depends on dissociation during capture and washing and must be measured for each workflow [20,21]. Further, data-dependent acquisition (DDA) can limit recovery by sampling the most abundant ions in each cycle [22]. Presentation predictors trained on recovered ligands can inherit these sampling biases [15]. Data-independent acquisition (DIA) improves sampling of the material reaching the instrument, but cannot recover peptides already lost during preparation [22]. Table 1 collects studies relevant to FDHS presentation and distinguishes measurements from inferences.

## 2. Complex Lifetime and Immune Recognition

Short-lived complexes can reach the cell surface and be recognised by T cells. Presentation abundance is the number of copies of a defined pMHC-I complex displayed per cell, and can be measured by a validated complex-specific reagent or by calibrated quantitative immunopeptidomics with a validated surface-specific isolation step [20]. Immunogenicity is the capacity of that complex to elicit a measurable T cell response under stated conditions. Immunodominance is the size of that response relative to others raised against the same pathogen or tumour. A functional response establishes that a ligand was displayed above the recognition threshold of the responding cells; it does not on its own measure copy number, peptide flux, or the off-rate of the complex. Most of the evidence below is immunological.

### 2.1. The Quantitative Range of pMHC-I Dissociation Rates

Reported values across pMHC-I complexes span a wide range of dissociation half-lives, from a few minutes to many hours [32,33,34]. For example, exogenously loaded H-2K^b^-restricted peptides on the surface of live cells treated with cytochalasin D to minimise endocytosis gave a *k*_off_ of 0.0495 h^−1^ for the ovalbumin-derived OVA-K^b^ complex (*t*_1/2_ ≈ 14.0 h) and 0.119 h^−1^ for the SIY model peptide–K^b^ complex (*t*_1/2_ ≈ 5.8 h) [32]. When considering peptide half-lives (calculated as *t*_1/2_ = ln(2)/*k*_off_), we are more concerned about the timescale of T cell surveillance, not the detection limits of mass spectrometry or any other assay. During contact with an antigen-presenting cell (APC), pMHC-I complexes with a half-life of one to five hours could remain available for recognition. Their abundance during that contact depends on continued delivery and the combined effects of dissociation and other routes of complex loss.

Analysis of a vaccinia peptide dataset found that pMHC-I stability predicted cytotoxic T cell immunogenicity better than equilibrium binding affinity [33,35]. Calis and colleagues also identified associations between immunogenicity and the properties and positions of amino acids exposed for T-cell receptor (TCR) contact [36]. Tapasin further favours presentation of peptides with longer pMHC lifetimes [4].

### 2.2. Unstable pMHC Complexes Can Elicit Potent CD8^+^ T Cell Responses

The clearest evidence comes from ERAAP-deficient cells, which lack a subset of conventional peptide–MHC complexes and display novel unedited ligands in their place. These novel ligands are short-lived, structurally unique complexes that elicit potent CD8^+^ T cell and B cell responses [9]. CD8^+^ T cells generated by immunising wild-type C57BL/6 mice with ERAAP-deficient spleen cells recognised these unedited ligands. Their stimulation fell by 80% after two hours of brefeldin A treatment, which blocks delivery of newly formed complexes to the surface. Stimulation of T cells specific for wild-type ligands was maintained over the same period [9]. Overall conventional pMHC-I expression declined by roughly 50% after four hours on both wild-type and ERAAP-deficient cells, so the instability of the novel ligands does not simply track bulk surface turnover [9].

ERAAP-deficient cells are sufficiently immunogenic to generate pMHC-I antibodies in MHC-matched hosts [9]. One such antibody, WEko.70, appears to recognise a structural feature shared by many ERAAP-unedited complexes [9]. These findings show that short-lived self-derived complexes can elicit immune responses [8,9,37].

The response in wild-type mice shows that tolerance to the conventional self-repertoire does not extend to all ERAAP-unedited ligands. The kinetic evidence is less direct: brefeldin A caused loss of T cell stimulation, which depends nonlinearly on ligand density, so the result cannot be converted into a peptide off-rate or an absolute surface half-life.

There is also evidence from productive immune responses to tumours. GSW11 is a cross-protective antigen from the murine colorectal cancer cell line CT26. GSW11 is encoded by the endogenous ecotropic murine leukaemia virus envelope gene and presented by H-2D^d^. After peptide loading of RMA-S cells, GSW11 stabilised surface H-2D^d^ with a half-life of approximately 20 min; this assay measured surface-complex loss, a surrogate for peptide dissociation [23].

This epitope is overtrimmed by ERAAP, so its generation rises when ERAAP is reduced [10]. BALB/c mice challenged with ERAAP-low CT26 developed protective CD8-dependent immunity that extended to rechallenge with wild-type CT26 [10]. Regulatory T cell depletion also revealed a strong GSW11-specific response [23]. GSW11-specific cells are also present in regulatory-T-cell-replete tumours, where many are functionally exhausted [24].

Experiments manipulating tapasin expression connected complex stability to antigen persistence. In a DNA-vaccination system that primed T cells through direct presentation by antigen-expressing cells, tapasin expression established response hierarchies according to pMHC stability [38]. This hierarchy was lost with a vaccine that relied on cross-presentation, in which presenting cells acquired antigen from other cells. Regulating transgene expression identified antigen persistence as a major determinant of the response. Together with the association between complex lifetime and immunogenicity in peptide panels [28], these findings show why supply, presentation route and stability must be considered together.

The ERAAP and GSW11 studies link altered processing to immune recognition [9,10,24]. In the ERAAP system, MS analysis of peptides eluted directly from the cell surface also identified an altered repertoire [39]. Connecting these findings to FDHS presentation requires quantifying how peptide supply and complex loss determine surface abundance, as illustrated in Figure 1.

### 2.3. Binding Properties and Recognition Thresholds

“Affinity”, “fast-dissociating”, “weak binder” and “stability” describe biochemical properties of receptor–ligand interactions and can refer to both the peptide–MHC and T-cell receptor (TCR)–pMHC interactions. However, the two can vary independently such that a peptide that binds MHC-I weakly can form a pMHC complex that binds tightly to a TCR. Equilibrium affinity describes binding strength, while kinetic stability describes how long a complex persists. For a single-step interaction the equilibrium dissociation constant is *K*_D_ = *k*_off_/*k*_on_, where *k*_off_ and *k*_on_ are the dissociation and association rate constants. A given *K*_D_ is therefore compatible with different off-rates. A half-maximal inhibitory concentration (IC_50_) measures competition under specified assay conditions and cannot be converted directly into *k*_off_ or a surface lifetime. Throughout this review, peptide off-rate refers to dissociation from MHC-I; dissociation of the TCR from pMHC is identified separately.

The question for a fast-dissociating peptide is whether enough copies are present during a T cell–APC contact, and the number required can be small. In a differentiated CTL clone already bound to its target, single-molecule labelling showed that three pMHC complexes at the contact interface sufficed for killing. Roughly ten were needed for a full calcium response and a mature immunological synapse [7]. These are counts at the contact interface and cannot be used as a universal whole-cell threshold. A single peptide–MHC class II ligand triggered cytokine secretion in naive, blast and memory CD4^+^ (cluster of differentiation 4-positive) T cells; additional ligands did not increase secretion per responding cell, an all-or-none or digital response [40]. This provides a separate example of how few ligands are needed for T cell activation.

Serial triggering, in which a small number of pMHC ligands successively engage multiple TCRs, provides one mechanism for sensitive recognition [41]. In the TCR–pMHC interactions examined by Huppa and colleagues, synaptic association and dissociation were both faster than in solution. The larger increase in association produced higher effective affinity, while actin disruption slowed dissociation [42]. Measurements in solution and at a membrane contact therefore describe different interaction conditions.

These experiments describe specific cytotoxic or cytokine responses and do not establish a universal threshold for productive naive CD8^+^ T cell priming by a dendritic cell. Priming also depends on costimulation, inflammatory signals and the duration of antigen presentation (Section 5.3 and Section 10.5) [43,44].

The surface pMHC copy number required to activate a T cell depends on its TCR and cellular state. Natural TCR–pMHC-I affinities lie mostly in the micromolar range and fall into distinct windows, with TCRs against human leukocyte antigen (HLA)-A2-restricted pathogen antigens binding roughly tenfold more tightly than TCRs against HLA-A2-restricted tumour-associated self-antigens [45]. The dataset is smaller but TCRs specific for neoantigens restricted by different HLA-I allotypes appear more similar in affinity to those recognising viral antigens [46]. These affinity differences help explain why the available TCR repertoire affects recognition [45,46], but they do not define a universal relation between affinity and the number of complexes required.

However, T cells with lower cellular avidity do not simply generate a weaker version of the same response. Here, cellular avidity refers to binding and functional measurements made on intact T cells, which are distinct from the affinity of one TCR for one pMHC. In the CT26 model, the efficacy of checkpoint blockade targeting programmed cell death protein 1 (PD-1) was associated with preferential expansion of low-avidity GSW11-specific CD8^+^ T cells. These T cells were more cytotoxic in vitro and controlled tumours better on adoptive transfer than the high-avidity population [25]. The study separated cells by tetramer-binding and functional properties; these measurements do not establish monomeric TCR affinity. In two experimental TCR systems, mutations that either shortened or prolonged TCR–pMHC binding reduced activation, supporting an optimal interaction duration under those conditions [47].

### 2.4. Slow Association and the Lifetime of Empty MHC-I

Successful loading requires a peptide to encounter receptive MHC-I before that molecule loses its binding competence. Eisen and colleagues estimated an association rate constant of approximately 4500 M^−1^ s^−1^ for exogenous OVA peptide binding to cell-surface H-2K^b^ [32]. Their model also identified the denaturation rate of empty MHC-I as an important determinant of loading. Because intracellular chaperones and the peptide-loading complex alter loading conditions in the ER, these surface-loading rates cannot be transferred to that compartment without measurement.

Experiments with H-2K^b^ support a two-step selection mechanism: initial binding admits a broad peptide pool, followed by faster dissociation of peptides with non-canonical binding features [31]. The process is strongly temperature-dependent. H-2K^b^ peptidomes from RMA cells cultured for three days at 26 °C versus 37 °C yielded 1381 and 553 peptides respectively, with only 341 shared, and peptides recovered at 26 °C were predicted to be weaker binders (median IC_50_ 33 nM versus 20 nM, *p* = 0.0009) and differed in sequence and length distribution [31]. The results support temperature-sensitive peptide selection, although temperature also affects cellular processing and transport [31].

The flexibility of the MHC-I peptide-binding groove also differs between allotypes, and it influences how stringently an allotype selects its cargo alongside peptide-binding specificity [48,49]. The temperature dependence also motivates a testable in vivo prediction that febrile temperatures of 39–40 °C may change the surface repertoire and the supply needed to maintain particular ligands. The direction and size of these effects require direct experimental measurements.

### 2.5. The Fate of MHC-I After Peptide Dissociation

Peptide dissociation can leave MHC-I in a peptide-receptive state, but the persistence of that state depends on the allotype and conditions. Reduced temperature stabilises empty MHC-I [50]. Structures of engineered, disulfide-stabilised HLA-A*02:01 reveal peptide-free and partially loaded conformations [51], while TAP-binding protein-related (TAPBPR) can promote peptide exchange in experimental systems [52]. These findings establish peptide receptivity and exchange under defined conditions, but leave the contribution of physiological surface reloading unquantified. Internalisation, recycling and loss of peptide receptivity must therefore be considered when interpreting surface-decay experiments.

## 3. A Model Relating Peptide Supply to Surface Complex Loss

The surface abundance of a defined pMHC-I complex is determined by both binding stability and peptide supply rate, which are distinct quantities linked by processing and loading. Peptide–MHC-I complexes with longer half-lives can accumulate at lower supply rates, while peptides that dissociate rapidly require greater delivery to maintain the same surface abundance (Figure 1). This relationship assumes comparable loading efficiency and other routes of surface loss. Recognition depends on the complexes available during T cell contact. A stable complex can remain available after peptide delivery stops, whereas a short-lived complex depends more strongly on continued delivery.

The model in Figure 1 separates two questions: how input and loss determine surface abundance, and whether that abundance supports recognition. Stability measurements address only part of the first question. The second also requires a cognate TCR repertoire and a defined cellular response, as the examples in Section 2 illustrate.

Binding predictors estimate peptide–HLA affinity, while eluted-ligand predictors estimate which peptides are likely to be recovered by immunopeptidomics. NetMHCpan-4.1 provides both outputs, and MHCflurry 2.0 combines binding with sequence-based processing predictions. NetMHCstabpan estimates complex half-life. Mechanistic models combine supply, peptide off-rate and competition during MHC-I loading to predict relative surface presentation [13,34,53,54,55,56]. The experimental challenge is to measure peptide input and successful delivery as rates per cell, then test how they determine absolute surface copy number. Section 10.2 sets out the quantities estimated by these approaches. The resulting predictions can then be tested against tumour presentation and T cell recognition.

Existing immunopeptidomics data are consistent with the trade-off between supply and binding affinity. In data from cells expressing single HLA-I allotypes, higher source-gene expression was associated with recovery of peptides with less favourable predicted HLA binding, so expression and affinity contributed jointly to the probability of observing a ligand [15]. Across HLA loci, recovered HLA-C ligands were associated with relatively high source expression and peptide hydrophobicity [17]. Structural and regulatory differences between HLA-C allotypes also affect their expression [57]. These associations concern recovered ligands and combine biological presentation with analytical detection; source expression alone cannot separate those contributions. Controlled experiments provide more direct evidence that peptide abundance, off-rate and tapasin expression jointly affect surface presentation [58].

Changes in peptide supply could influence how both T cells and natural killer (NK) cells recognise HLA-C. Its surface expression is generally lower than that of HLA-A and HLA-B in the cells studied [59]. It also has a central role in regulating NK cells through the ‘missing-self’ response and NK cell education via inhibitory killer-cell immunoglobulin-like receptor (KIR) interactions [60]. As KIR binding to HLA-I is dependent on both HLA-I surface level and the peptide sequence [61,62], the two-axis model could have consequences for both adaptive and innate immunity. HLA-C expression also depends on messenger RNA (mRNA) turnover and allotype-specific peptide-binding properties, which must be distinguished from peptide supply [57,63].

### A Minimal Steady-State Model

The relationship between peptide supply and complex loss follows the peptide filtering relation, which links surface abundance to supply, unbinding and tapasin [53]. Let *S* be the rate at which copies of a candidate sequence enter processing within peptide-containing substrates, in molecules per cell per hour. Let *φ* be the fraction delivered as new surface complexes after generation, destruction, transport, trimming, competition, loading and export. The delivery rate is *J* = *Sφ*.

Let *k*_off_ and *k*_other_ denote the peptide dissociation rate and the rate of other surface losses, chiefly internalisation and turnover, both in h^−1^. Loading, editing and the loss of empty-MHC receptivity contribute to *φ*. If delivery is constant and losses are proportional to surface copy number *N*, d*N*/d*t* = *J* − (*k*_off_ + *k*_other_)*N*, where *t* is time in hours. At steady state:

*N*_ss_ = *Sφ*/(*k*_off_ + *k*_other_)

Under the fixed-yield approximation, the fraction *φ* delivered to the surface remains constant as input changes. When peptide dissociation dominates surface loss, *N*_ss_ is approximately *Sφ*/*k*_off_. For illustration, *S* = 500 molecules cell^−1^ h^−1^, *φ* = 0.20, *k*_off_ = 0.5 h^−1^ and *k*_other_ = 0.10 h^−1^ give about 167 surface complexes. Increasing *k*_off_ to 5 h^−1^ reduces the count to 19.6 if all other quantities remain fixed. Raising *S* to 4250 molecules cell^−1^ h^−1^ restores the original count. The required 8.5-fold increase follows from the ratio of total loss rates, 5.1/0.6. When *k*_off_ is much smaller than *k*_other_, further reductions in dissociation have progressively less effect because other losses dominate.

Holding *φ* fixed isolates the effect of surface loss. In cells, *φ* may fall for a rapidly dissociating ligand because it is also retained less efficiently during loading and export. If so, the required increase in supply would exceed the ratio of surface loss rates. Testing the numerical prediction requires calibrated surface counts; relative MS signals across viral ligands [64] cannot establish those counts. Recognition must then be measured separately, because a whole-cell count does not specify how many ligands a T cell encounters [7].

Many allotypes may exhibit a supply-dependent threshold for marginal ligands under appropriate loading conditions. There are multiple reasons why some peptides will not reach the surface despite increased supply, including when they are incompatible with binding a specific HLA-I allotype. Other reasons include slow association with receptive MHC-I, destruction by cytosolic or ER aminopeptidases before loading, and removal during peptide editing. The supply needed for recognition is a property of a peptide–allotype pair in a given cellular context, not of the allotype alone. Mutant KRAS (Kirsten rat sarcoma viral oncogene homologue) in Section 6 illustrates the importance of binding compatibility: the G12D substitution creates an anchor interaction with HLA-C*08:02 that the tested wild-type peptides lack [65].

Thousands of peptides compete for a finite loading system, and tapasin favours complexes with longer lifetimes [4,5,53,58]. Mechanistic models describe this competition [13], which is absorbed into the delivery rate *J* in the single-peptide model. Its predictions therefore depend on measuring or adequately approximating *J*.

Before steady state, the same balance gives *N*(*t*) = *N*_ss_ + [*N*(0) − *N*_ss_]exp[−(*k*_off_ + *k*_other_)*t*]. After delivery is completely blocked, the corresponding decay is *N*(*t*) = *N*(0)exp[−(*k*_off_ + *k*_other_)*t*]. A calibrated surface-decay experiment therefore measures total loss under these assumptions.

Experiments varying peptide abundance and off-rate have measured relative surface presentation [13,58]. Table 1 summarises complementary evidence, with immunological readouts establishing recognition in some systems. For endogenous FDHS ligands, the proposed quantitative compensation remains to be tested using independently measured input, delivery, loss and surface copy number in one system (Section 9).

Peptide supply and pMHC-I stability jointly determine the surface abundance shown in Figure 1. We calculate surface pMHC-I counts to show how changes in supply offset differences in pMHC-I dissociation rates (Figure 1A). We highlight where the same surface pMHC-I count can be achieved by fast-dissociating peptides with additional supply input and where slowly dissociating ones can do without additional supply. We predict that increasing antigen production could increase the surface abundance of a short-lived complex. The required input increase follows total surface loss, so the example needs 8.5-fold more input to offset tenfold faster dissociation, with other parameters fixed (Figure 1B).

## 4. Mechanisms Determining Peptide Supply

Peptide supply is not determined by the steady-state abundance of source proteins alone. Evidence from the study of defective ribosomal products, ribosome-associated quality control, and quantitative immunopeptidomics shows that synthesis, degradation and source-protein abundance all contribute to presentation [29,66,67,68] (Table 2).

### 4.1. Defective Ribosomal Products

Yewdell, Antón and Bennink proposed in 1996 that a significant fraction of ‘self’ and viral peptides derive from defective ribosomal products (DRiPs), explaining how viral peptides appear on infected cells so quickly [69]. DRiPs include prematurely terminated and misfolded polypeptides produced during protein synthesis. Dynamic stable isotope labelling by amino acids in cell culture (SILAC) showed that isotope incorporation into many MHC-I-bound peptides is faster than into their source proteins, consistent with a contribution from rapidly degraded newly synthesised products [66]. Some peptides derive from proteins undetectable by standard proteomics [2].

Calculations of DRiP-derived supply depend on the fraction of newly synthesised protein that is rapidly degraded. For a hypothetical epitope supplied entirely by this route, assume that a 30% rapidly degraded fraction produces 100 surface complexes. Reducing that fraction to 5% would lower the predicted count to about 17, provided that synthesis and all other delivery and loss parameters remain fixed. Contributions from mature-protein turnover would need to be added separately. These fractions illustrate sensitivity to an uncertain parameter; the early estimate of approximately 30% [70] is not a universal cellular value [71]. Quantitative estimates of the DRiP contribution are challenging, creating uncertainty in applications of the model. Estimating the supply axis therefore needs a synthesis rate, a degradation fraction and an epitope yield measured in the system under study.

The mode of antigen presentation affects the supply axis, while the intrinsic stability of a given peptide–MHC complex is comparable only under matched conditions. For example, direct presentation can draw on continuous intracellular synthesis. During cross-presentation, supply depends on antigen uptake and persistence, including repeated uptake or intracellular storage [43,44]. When an antigen is available only as a brief pulse, the off-rate may become limiting, since a short-lived complex formed during a brief processing window can be gone before the dendritic cell (DC) meets a naive T cell (Section 5.3 and Section 10.5). These effects also depend on the antigen-presenting cell type and activation state.

### 4.2. Ribosome-Associated Quality Control and Tumour Context

Ribosome-associated quality control (RQC) connects failed translation to antigen processing by resolving stalled translation complexes and disposing of incomplete nascent proteins. Stalled ribosomes are split, and the incomplete nascent chain is ubiquitinated by Ltn1/Listerin and extracted for proteasomal degradation [80,81]. Entry into this route does not require a completed source protein. Listerin perturbation changes presentation of a subset of peptides, directly supporting an RQC contribution to the immunopeptidome [29].

Tumour-associated changes in translation and protein handling make RQC a candidate source of altered antigen supply. ER stress can also suppress translation or impair presentation, so its net effect must be resolved for individual ligands [76,77,78,79]. Comparing tumour and normal cells therefore requires measurements of substrate production, processing yield and surface presentation. For the tolerance hypothesis, the further question is whether the relevant substrates are generated and presented during thymic selection.

### 4.3. Distinguishing Peptide Supply from Analytical Recovery

Immunopeptidomics measures peptides recovered through a particular workflow. Biological losses occur before surface delivery through peptide destruction, inefficient transport or unsuccessful loading. Analytical losses occur during sample preparation, acquisition and identification (Figure 2A). DIA can improve detection of low-abundance peptides in an extract, but it cannot recover material lost before acquisition [22]. Shorter capture and washing steps could improve retention of labile complexes, although this must be tested against changes in purity and total yield. Surface acid elution, which recovered ERAAP-dependent and ERAAP-independent ligands [39], provides a complementary sampling method with its own recovery limitations. Separating these effects is necessary before additional peptide identifications can be interpreted as evidence for short-lived surface complexes (Figure 2B).

## 5. Antigen Presentation and Viral Immunodominance

Evidence from viral systems shows that pMHC stability alone does not predict the magnitude of the CD8^+^ T cell response. Antigen availability, the cells presenting it and the responding T cell population also influence the hierarchy [11,12,14,33,83].

### 5.1. Influenza A NP366–374

During primary influenza infection in C57BL/6 mice, PA224-specific CD8^+^ T cells are at least as prominent as NP366–374-specific cells during the first ten days in the bronchoalveolar lavage population studied. During secondary influenza responses, NP366-specific cells are more numerous than PA224-specific cells [11]. In a separate surface-decay experiment after peptide loading and brefeldin A treatment, D^b^/NP366 had a shorter measured half-life than D^b^/PA224, approximately 223 versus 408 min [13]. That experiment measured loss of surface complexes, and the primary-to-secondary hierarchy cannot be assigned to a difference in peptide dissociation alone. The size of these responses has pathological consequences, since influenza-specific CD8^+^ T cells contribute to lung immunopathology as well as to clearance [84].

One interpretation links the reversal of immunodominance to the distribution of antigen presentation across cell types. In the tested cells, efficient PA224 presentation was largely restricted to DCs, while NP366 was presented by both dendritic and non-dendritic cells [12]. Naive T cells depend on competent priming cells, whereas memory T cells can respond to a broader range of presenting cells. This difference offers an explanation for the primary and recall hierarchies.

Dissection of the H-2K^d^-restricted influenza response also showed that presentation abundance alone does not determine immunodominance. Inefficient processing explained the poor immunogenicity of one subdominant epitope, while overexpression failed to overcome limitations in responses to two others. Those limitations were unrelated to TCR diversity or sensitivity in the assays used [83]. Responses to individual epitopes also differed in their capacity to suppress responses to other epitopes, a process termed immunodomination. Structural work on human influenza epitopes further shows how TCR recognition constrains the response [85]. These studies identify distinct contributions from processing and T cell biology.

### 5.2. LCMV GP276–286

During chronic LCMV infection with Clone 13, T cells specific for four epitopes with different affinity values have distinct fates. The normally dominant NP396-specific T cells are deleted, while GP33-specific cells persist but become exhausted and GP276-specific cells become prominent but also show functional impairment; GP34-specific cells are deleted [14]. The epitopes recognised by the deleted populations, NP396 and GP34, showed greater relative presentation in a T cell proliferation assay and bound MHC-I with IC_50_ values of 4.4 and 22 nM. GP33 and GP276, whose responding cells persisted, showed lower relative presentation and IC_50_ values of 775 and 52 nM [14]. These values came from competition assays and do not establish complex lifetimes. The study links persistent antigen exposure to deletion and exhaustion.

For FDHS peptides, these results require presentation and protective function to be assessed separately. Even if high supply maintains a short-lived ligand, persistent stimulation can accompany dysfunction or deletion of the responding T cells.

### 5.3. HIV Gag-Derived Epitopes

Studies of T cell responses to a human immunodeficiency virus (HIV) group-specific antigen (Gag) illustrate why the timing and route of antigen supply matter. Gag is the precursor of major virion structural proteins. In a cohort of untreated people with HIV, Gag-specific responses were associated with lower viraemia, while responses to several other viral proteins were associated with higher viraemia [86]. Incoming virion proteins can supply peptides before de novo viral protein synthesis. In the simian immunodeficiency virus (SIV) system, Gag-specific CD8^+^ T cells recognised infected CD4^+^ T cells as early as two hours after infection, before integration and new viral protein synthesis [87]. This demonstrates rapid presentation from incoming material, which is a finite source unless replenished. Later direct presentation can draw on newly synthesised Gag.

For dendritic cells, direct presentation draws on intracellular antigens, while cross-presentation depends on the uptake and persistence of material acquired from other cells [43,44]. Tests of off-rate must therefore distinguish these routes and measure priming separately from effector recognition.

SL9 (SLYNTVATL, Gag p17 residues 77–85) elicits prominent HLA-A2-restricted responses during chronic HIV infection [27]. In vitro-primed SL9-specific T cells also sustained expansion without CD4^+^ cells or exogenous interleukin-2, supported by autocrine mediators in the cultures studied [88]. Structural and functional analysis of SL9 escape variants showed contributions from reduced pMHC stability and altered TCR recognition [26]. The affinity measured for the 868 TCR was approximately 82 nM, yet strong receptor binding did not prevent escape when effective ligand availability was reduced. This illustrates why pMHC stability and TCR binding must be considered separately.

HLA-B*57:01, an allotype associated with HIV control, is highly tapasin-dependent [89]. Tapasin dependence provides a further variable when comparing presentation across allotypes, because editing can enrich stable complexes and change repertoire breadth (Section 7). This association does not identify the protective mechanism.

## 6. Oncoproteins as Candidate Sources for Supply Measurements

Oncoproteins provide candidate systems for testing whether high antigen production can maintain short-lived surface complexes. High synthesis accompanied by rapid degradation can increase substrate flow through the proteasome, but increased steady-state protein abundance may instead result from reduced degradation. Table 3 distinguishes these possibilities for c-Myc, Cyclin D1, β-catenin and mutant KRAS.

An illustrative turnover calculation shows why source-protein abundance must be separated from epitope delivery. A pool of 10,000 protein molecules with a 25 min half-life has an instantaneous first-order degradation rate of approximately 16,600 molecules per hour. Sustaining that rate requires synthesis to replenish the pool; without replenishment, the pool and degradation rate decline. This is a calculated source-protein flux, not a measurement of Myc abundance or peptide production. The fraction yielding a particular pMHC must be measured independently. A bulk antigen-processing efficiency from another protein or cell system [67] cannot establish the number of Myc-derived complexes, their detectability by MS or their capacity to activate T cells.

Mutant KRAS also illustrates two distinct mechanisms by which mutations generate neoantigens. Some mutations occur at non-anchor positions in peptides already able to bind the host HLA molecule. These can alter TCR contacts or peptide conformation while preserving broadly similar HLA binding. Other mutations create or strengthen an HLA anchor, allowing a previously poorly presented sequence to form a stable complex [6,46]. KRAS-G12D is an example of the latter mechanism for HLA-C*08:02: the mutant aspartate forms a salt bridge with Arg156, and the corresponding wild-type peptides failed to stabilise HLA-C*08:02 in the reported assays [65]. Supply remains relevant once a compatible ligand is generated, but increased expression cannot be assumed to rescue a missing anchor interaction.

These oncoproteins provide systems in which to measure the proposed compensation. Pulsed-SILAC can compare source turnover with peptide appearance in selected tumour models [66]. Combining those data with surface-specific quantification and direct dissociation measurements would establish whether a particular ligand is maintained by high input.

## 7. Tapasin Editing and Peptide Supply

Tapasin influences which peptides survive loading and reach the cell surface. MHC-I assembly proceeds through peptide-binding and quality-control steps [97], and tapasin promotes exchange and enrichment of more stable cargo [4,5]. The effect depends on the peptide and MHC-I allotype. This provides a mechanism by which peptide input and loading efficiency interact before surface loss becomes relevant.

Boulanger and colleagues varied expression of fluorescent ubiquitin–peptide constructs and compared presentation in tapasin-sufficient and tapasin-deficient cells [58]. The benefit of tapasin depended on peptide abundance and the previously measured off-rates of peptide variants. High expression also supported the presentation of lower-affinity variants in tapasin-deficient cells. These findings directly link input, binding and editing. The antigen-binding fragment (Fab) used to detect the complexes bound the variants with different affinities, so staining intensities could not be compared as absolute copy numbers across variants. Quantifying compensation across variants requires separate calibration of the detection reagent for each complex.

The C57BL/6 infection studies use a fixed classical MHC-I background, H-2K^b^ and H-2D^b^. Human HLA-I diversity introduces additional variation in peptide selection and tapasin dependence [89]. The relationship between supply, editing and recognition therefore needs validation across defined human allotypes.

Immunopeptidomes from different HLA-I allotypes also differ in how much tapasin-dependent editing they need. HLA-B*27:05 achieves high surface expression and presents viral peptides in the complete absence of tapasin, while the tapasin-deficient cells display more HLA-B27 free heavy chains. Adding exogenous peptide lowers this free-heavy-chain signal. This is consistent with less stable loading, although it does not directly measure peptide off-rates [98]. HLA-B*44:05 is tapasin-independent while the nearly identical HLA-B*44:02, differing at position 116, is tapasin-dependent [5]. The basis for this difference is conformational: HLA-I allotypes differ in the intrinsic plasticity of their peptide-binding domains, which influences how effectively an allotype selects stable peptides without tapasin, alongside its peptide-binding specificity [48,49,99].

Tapasin-independent allotypes were associated with broader HIV epitope responses, and genotypes with greater tapasin independence were associated with lower viral loads and slower disease progression [89]. These observations support repertoire breadth as a possible link to control, without proving that it mediates the clinical association. Tapasin dependence and HLA-I zygosity can each influence repertoire breadth [89].

Tapasin can narrow the repertoire as well as favouring more stable complexes, focusing the immunopeptidome onto a smaller set of peptides in the systems studied [100]. The effect of tapasin loss depends on the allotype and ligand. In a study of 85 non-small cell lung cancers, tapasin expression correlated with survival and CD8^+^ T cell infiltration. Experimentally generated tapasin-deficient lung and colon cancer variants escaped recognition by CTLs specific for endogenous tumour antigens despite retaining antigen expression [101]. TAPBPR also mediates peptide exchange and has allotype-specific preferences [49,102]. Its contribution cannot be assumed to compensate fully for tapasin loss.

Tapasin loss does not appear therapeutically favourable in the tumour models studied [101]. It impaired recognition of the tested endogenous antigens, so a broader repertoire cannot be equated with better immune control. The framework can be tested in both tapasin-sufficient and tapasin-low tumours, with delivery efficiency and overall MHC-I expression measured in each. High supply, reduced editing or a tapasin-independent allotype may permit presentation of some less stable ligands, but none guarantees successful loading or immune recognition [58,89,100].

## 8. Non-Canonical Translation Products and the Expanded Peptidome

Several routes generate MHC-I ligands absent from databases of annotated protein sequences. Binding predictors can score many such sequences once they are supplied, but they cannot nominate sequences missing from the candidate set. For each route, transcript expression and translation determine substrate input, while proteolysis, modification and loading influence the yield of individual ligands. Non-canonical ligands have been identified in malignant cells [82,103,104], although their origin alone does not specify pMHC stability. Table 4 compares these sources with other changes in antigen processing and identifies the measurements needed to relate their products to FDHS peptide presentation.

Cryptic peptides from non-canonical reading frames expand the candidate antigen repertoire [82,103,104]. A peptide from a downstream open reading frame of ATP-binding cassette subfamily B member 5 (ABCB5) was identified by MS and elicited interferon-γ (IFN-γ) responses from autologous tumour-infiltrating and circulating CD8^+^ T cells after peptide stimulation [82]. ABCB5 transcripts were detected in 37% of the melanoma cells examined. These results establish peptide recognition and heterogeneous source expression, but do not demonstrate killing of unpulsed tumour cells [82].

The immunoproteasome changes the yield and identity of generated peptides [109]. A different peptide repertoire can have a different distribution of pMHC stabilities, but altered proteasomal processing does not itself change the intrinsic dissociation rate of a given peptide–MHC pair under matched conditions. IFN-γ also induces ERAP1/ERAAP, whose effect depends on the context. In the human cell systems examined by York and colleagues, ERAP1 knockdown increased overall surface HLA-I under basal conditions but reduced it after IFN-γ treatment. The authors linked this change to increased generation of N-terminally extended precursors requiring trimming; purified ERAP1 could also destroy some nine-residue epitopes by further trimming [37]. These opposing effects make precursor sequence and inflammatory state relevant to processing predictions. Several allotypes present eight-residue peptides efficiently, so the effect of further trimming must be established for each peptide–allotype pair.

## 9. Experiments to Test Supply Compensation

Table 5 sets out seven predictions and the experiments that test them. The first prediction is that increased peptide input can offset faster surface-complex loss when delivery efficiency remains constant. The remaining six experiments examine analytical recovery, multimer stability, source turnover, peptide editing and temperature-dependent loss, at different steps in presentation or measurement.

A direct test of the first prediction uses graded, inducible expression of a defined antigen and peptide variants with independently measured complex loss rates. Estimating input and delivery efficiency before measuring surface counts allows the predicted compensation to be tested without fitting these parameters to the outcome. Recognition assays with characterised cognate T cells then determine whether the maintained complexes remain functional targets.

Antigen induction must not unintentionally alter MHC-I expression, interferon exposure, viability or trafficking. Surface reagents must be calibrated separately for each variant, because different binding affinities produce different signals per complex. Whole-cell immunopeptidomics requires a validated surface-specific separation before it can provide surface counts. The prespecified delivery efficiency, *φ*, must not be adjusted to fit the observed surface count. A failure to maintain presentation despite the prespecified input increase would challenge the fixed-yield approximation in that range. Failure to elicit recognition at the maintained density would challenge the proposed functional benefit.

A second prediction is that some short-lived ligands are lost during sample preparation. A direct test would compare standard and shorter capture workflows on matched samples while holding the acquisition and search procedures constant. Improved recovery of independently validated, rapidly dissociating complexes with shorter capture would support this explanation. Crosslinking could be tested as a separate intervention, with controls for altered peptide recovery and identification. The expected effect size should be specified from measured handling times, complex lifetimes and assay sensitivity before analysing the comparison.

The reagent tests need particular care because tethering a peptide can alter pMHC conformation. Additional multimer staining is useful only if the detected T cells recognise a naturally processed antigen. GSW11 single-chain reagents and tapasin perturbations provide experimental precedents [24,25,58,100]; Table 5 specifies the additional measurements needed to connect each intervention to the FDHS hypothesis.

Binding affinity, predicted rank and measured complex half-life are continuous quantities, even when candidate-selection workflows apply cutoffs. The proposed experiments should retain those measurements and report their uncertainties, alongside input and surface counts. A binary binder classification alone cannot establish the relationship between peptide delivery and loss. Functional assays are then needed to determine whether any increase in presentation changes recognition.

## 10. Implications

### 10.1. Neoantigen Discovery and Immunotherapy

Some neoantigen discovery workflows filter candidates by predicted binding affinity, for example, using an IC_50_ threshold of 500 nM [6]. Such filtering can exclude recognised ligands, but affinity is not a direct measure of dissociation and expression alone does not identify FDHS peptides. GSW11 provides a related example of a tumour-associated retroviral antigen. Reducing ERAAP-mediated destruction increases its presentation and induces protective CD8-dependent immunity against CT26 [10], while regulatory T cell depletion exposes a cross-protective response that includes GSW11-specific cells [23]. Incorporating source-protein expression and turnover into prioritisation should be tested against existing expression-aware models [15] and natural tumour presentation. For candidates generated selectively in tumour conditions, thymic presentation provides an additional criterion: limited exposure during selection could leave a more responsive T cell repertoire (Section 10.3).

Investigations into the LCMV GP33 ligand illustrated the limitation of a universal affinity cutoff. Its reported IC_50_ was 775 nM, above the commonly used 500 nM threshold, yet GP33-specific T cells responded to infection [14]. This is evidence about candidate filtering, not a measurement of peptide dissociation or preserved function during chronic infection.

Loss of heterozygosity at the HLA-I locus was reported in approximately 40% of the non-small cell lung cancers examined in one study [110]. This eliminates presentation by the lost allele and can remove peptides that are not also presented by retained alleles. The reduction in repertoire diversity depends on which alleles are lost and on how far their repertoires overlap [110]. Somatic mutations in HLA-I genes provide another route to altered antigen presentation [111]. HLA-I genotype was also associated with survival after immune checkpoint blockade [112]. Personalised cancer vaccines may benefit from selecting epitopes that reflect the natural presentation dynamics of the tumour, shaped by supply as well as by binding stability.

During immunoediting, immune pressure can favour tumour cells that escape recognition. Tumours can evade T cell recognition through antigen loss, reduced transcription of antigen-presentation genes, HLA-I loss of heterozygosity, impaired antigen processing and defects in interferon signalling [79,110,111,113]. Recognition also depends on the available T cell repertoire [45,46]. These studies do not establish whether the epitopes presented on tumours resulting from immunoediting are enriched for targets with moderate or weak MHC-I binding. Enrichment for FDHS epitopes is a conditional hypothesis: it would require immune selection to remove targets preferentially according to complex stability and recognition while preserving adequate supply of the remaining ligands. Stable complexes that are not recognised could also remain. Matched measurements before and after immune selection could test for enrichment, with peptide input and complex lifetime measured separately.

A neoantigen fitness model combining binding and T cell recognition features predicted survival after immune checkpoint blockade without incorporating source-protein expression or turnover [114]. Adding independently measured source dynamics would test whether supply improves prediction beyond binding and recognition features.

Vaccination changes antigen availability, with the delivery platform determining where, how much and for how long antigen is produced or acquired. Experiments with regulated DNA-vaccine expression show that antigen persistence can influence response hierarchies [38]. Because antigen production and processing in vaccine-presenting cells can differ from those in the tumour, the relevant therapeutic endpoint is recognition of naturally processed tumour antigen at its endogenous surface density.

### 10.2. Computational Prediction

Epitope prediction tools can combine measurements of antigen production and processing to account for the supply axis. Source-protein expression and turnover inform supply. Complementary inputs include pMHC off-rate, equilibrium binding affinity, HLA-I allotype-specific tapasin dependence and processing predictions appropriate to the inflammatory context. Ribosome profiling and pulsed-SILAC provide information about translation and turnover, but do not alone measure the epitope-specific DRiP contribution. The model relates these measurements to peptide input, successful delivery and complex loss. A neural network integrating binding motifs, source gene expression, and proteasomal cleavability improved prediction of recovered HLA ligands compared with models trained on binding-affinity data [15]. Integrating structural, computational and immunological evidence can help establish where mechanistic models complement sequence-based predictors [115]. Table 6 summarises the endpoints estimated by these approaches.

A proposed model should be benchmarked against existing expression- and processing-aware approaches using independent data. Peptide recovery, absolute surface presentation and T cell recognition are separate endpoints, and improvements must be validated for each.

Predicting longer or shorter peptides requires attention to allotype-specific length preferences and the representation of each length in training data. Many HLA-I immunopeptidomes are dominated by nine-residue peptides. NetMHCpan-4.1 accommodates variable lengths through binding-core alignment, including insertions and deletions [54] (Table 6). Peptides longer than nine residues can bulge out of the groove or extend beyond its ends [119,120]. Epitope mapping has historically emphasised the common nine-residue length [121], and peptide length can itself change TCR engagement [122]. Performance should therefore be evaluated by peptide length and allotype before changing selection thresholds.

Stability predictions from tools such as NetMHCstabpan address a different endpoint from equilibrium binding and require their own validation [34]. Agreement between predictors can guide prioritisation, but correlated errors mean that it cannot substitute for experimental confirmation.

### 10.3. Central Tolerance and Tumour-Selective Recognition

Central tolerance removes developing T cells that recognise self-antigens strongly in the thymus, shaping the repertoire available for tumour recognition [46]. We propose that tumour-associated translational stress can sustain peripheral presentation of some short-lived ligands that were poorly displayed during thymic selection. If the processes generating a ligand are absent or weak in the thymus, limited presentation could spare responsive T cells. The resulting FDHS target would combine sustained tumour presentation with reduced constraint from central tolerance.

The relevant comparison is between antigen presentation in the thymus and the tumour. The thymoproteasome shapes peptide generation in cortical thymic epithelial cells and supports CD8^+^ T cell development [123]. Its role in positive selection does not by itself establish escape from negative selection. Indeed, immunopeptidomics has identified tumour-associated peptides in a healthy human thymus, including on dendritic cells involved in negative selection [124].

Translational stress provides a mechanism for generating a different tumour repertoire. In melanoma cells, prolonged IFN-γ exposure induced indoleamine 2,3-dioxygenase 1 (IDO1), depleted tryptophan and promoted ribosomal frameshifting. Aberrant peptides were recovered from HLA-I complexes, and peptide-pulsed dendritic cells primed peptide-specific CD8^+^ T cells from healthy donors [125]. This connects stress-dependent peptide production to an available T cell repertoire, although the study did not measure thymic presentation or pMHC dissociation. These peptides are candidates for testing the proposed link between FDHS presentation and central tolerance.

Stress-associated origin alone is insufficient to infer escape from tolerance. In mouse models, thymic innate-like T cells directly presented a model inflammation-associated antigen and efficiently deleted cognate CD8^+^ thymocytes [126]. The predicted advantage therefore belongs to FDHS ligands whose thymic presentation is insufficient for deletion. Their presentation in tumours would depend on the increased input described by the supply model, while the responding repertoire would reflect the earlier conditions of thymic selection.

This hypothesis can be tested in a defined peptide–MHC model by changing thymic antigen supply and measuring deletion of cognate T cells while holding tumour antigen expression constant. Matched tumour experiments should establish rapid dissociation, compensation by input and recognition of naturally processed antigens. Healthy cells must also be examined under both basal and relevant stress conditions, because limited thymic presentation does not establish tumour specificity. Transcript abundance and failure to detect a peptide by MS are useful screening observations, but neither alone demonstrates absence during thymic selection.

### 10.4. NK Cell Recognition

NK cells can respond to changes in HLA-I abundance and peptide cargo through inhibitory receptors, so altered peptide supply could influence innate recognition as well [60,61,62,127]. KIR interactions with classical HLA-I and CD94 (cluster of differentiation 94)/NKG2A (natural killer group 2 member A) interactions with HLA-E must be assessed separately because they recognise different ligand sets.

### 10.5. Antigen Transfer and CD8^+^ T Cell Priming

For CD8^+^ T cell priming, antigen transfer to dendritic cells adds another step between source production and surface presentation. Autophagy delivers cellular material to lysosomes for degradation and participates in several antigen-processing pathways [128]. Endogenous autophagic processing of a viral nuclear antigen was demonstrated for MHC class II [129]. Autophagy in tumour cells also promoted antigen cross-presentation by recipient dendritic cells [130]. The latter finding concerns antigen transfer and does not establish a proteasome- or TAP-independent loading route in the donor tumour cell.

Priming by a fast-dissociating ligand requires sufficient pMHC to remain on the dendritic cell when a cognate naive T cell arrives. Repeated antigen uptake or intracellular retention could prolong delivery after the initial exposure [43,44].

## 11. Conclusions

The FDHS framework asks how rapidly dissociating peptides remain available for immune recognition when supply is high. Peptide production, successful loading and surface complex loss determine that availability. Short-lived complexes can elicit immune responses, and experiments manipulating peptide abundance and tapasin establish effects of input and editing on relative presentation. The remaining quantitative question is how often endogenous tumour ligands depend on this compensation and how well current methods recover them.

The two-axis framework organises existing observations by relating surface abundance to peptide delivery and complex loss. Existing experiments and mechanistic models already connect peptide abundance, binding and editing. The proposed tests ask how far this relationship extends to endogenous FDHS ligands when input, delivery efficiency, surface loss and absolute copy number are independently measured.

The tolerance hypothesis adds a reason to pursue FDHS targets generated selectively under tumour conditions: limited thymic presentation could preserve responsive T cells while increased tumour supply sustains their target. Testing that link requires evidence for both presentation and repertoire availability. Failure of a prespecified input increase to restore surface abundance would challenge the fixed-yield approximation in the tested conditions; restored abundance without recognition would leave the proposed functional benefit unsupported. Together, these tests can determine whether FDHS presentation identifies tumour targets missed by stability-led selection.

## Figures and Tables

**Figure 1:**
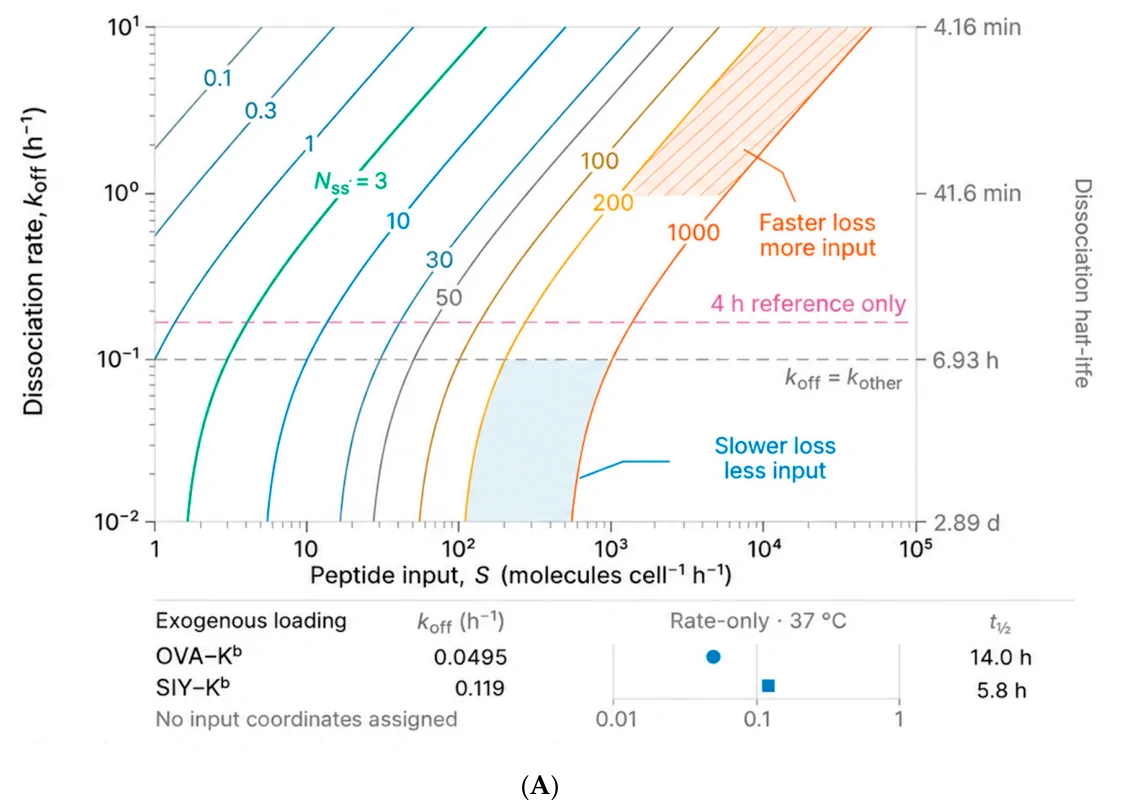

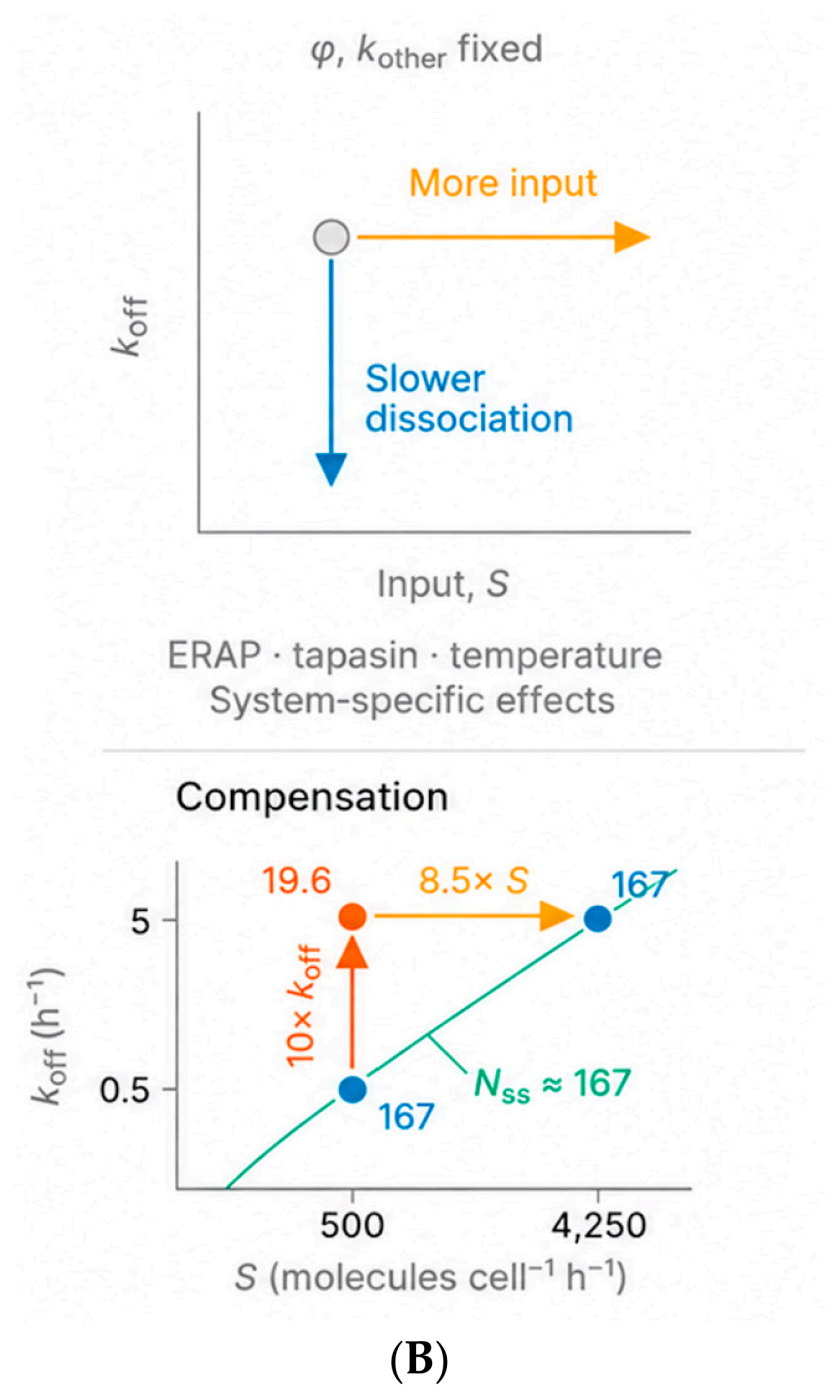
The two-axis model for cell-surface peptide–MHC-I complex abundance. (**A**) Contours show *N*_ss_ = *Sφ*/(*k*_off_ + *k*_other_), with illustrative values *φ* = 0.20 and *k*_other_ = 0.10 h^−1^. *N*_ss_ is the steady-state surface complex count; *k*_off_ is the peptide dissociation rate and *k*_other_ is the rate of other surface losses. *S* is the rate of candidate-sequence input into processing; *φ* is the fraction delivered as surface complexes. The right axis gives dissociation half-life, *t*_1/2_ = ln(2)/*k*_off_. The green contour marks three whole-cell complexes for numerical reference. It is not a recognition threshold: Purbhoo and colleagues counted three ligands at an established T cell contact interface [7]. The four-hour line is also a reference. Coloured bands illustrate comparable counts at different input and loss rates. The inset gives exogenous-loading rates for OVA–H-2K^b^ and SIY–H-2K^b^ without assigning inputs [32]. (**B**) With *φ* and *k*_other_ fixed, increasing *k*_off_ from 0.5 to 5 h^−1^ at *S* = 500 molecules cell^−1^ h^−1^ lowers *N*_ss_ from 167 to 19.6. Increasing *S* to 4250 restores the original count. These are model calculations, not endogenous measurements. OVA, ovalbumin-derived peptide; SIY, SIYRYYGL model peptide; ERAP, endoplasmic reticulum aminopeptidase. MHC-I, major histocompatibility complex class I.

**Figure 2:**
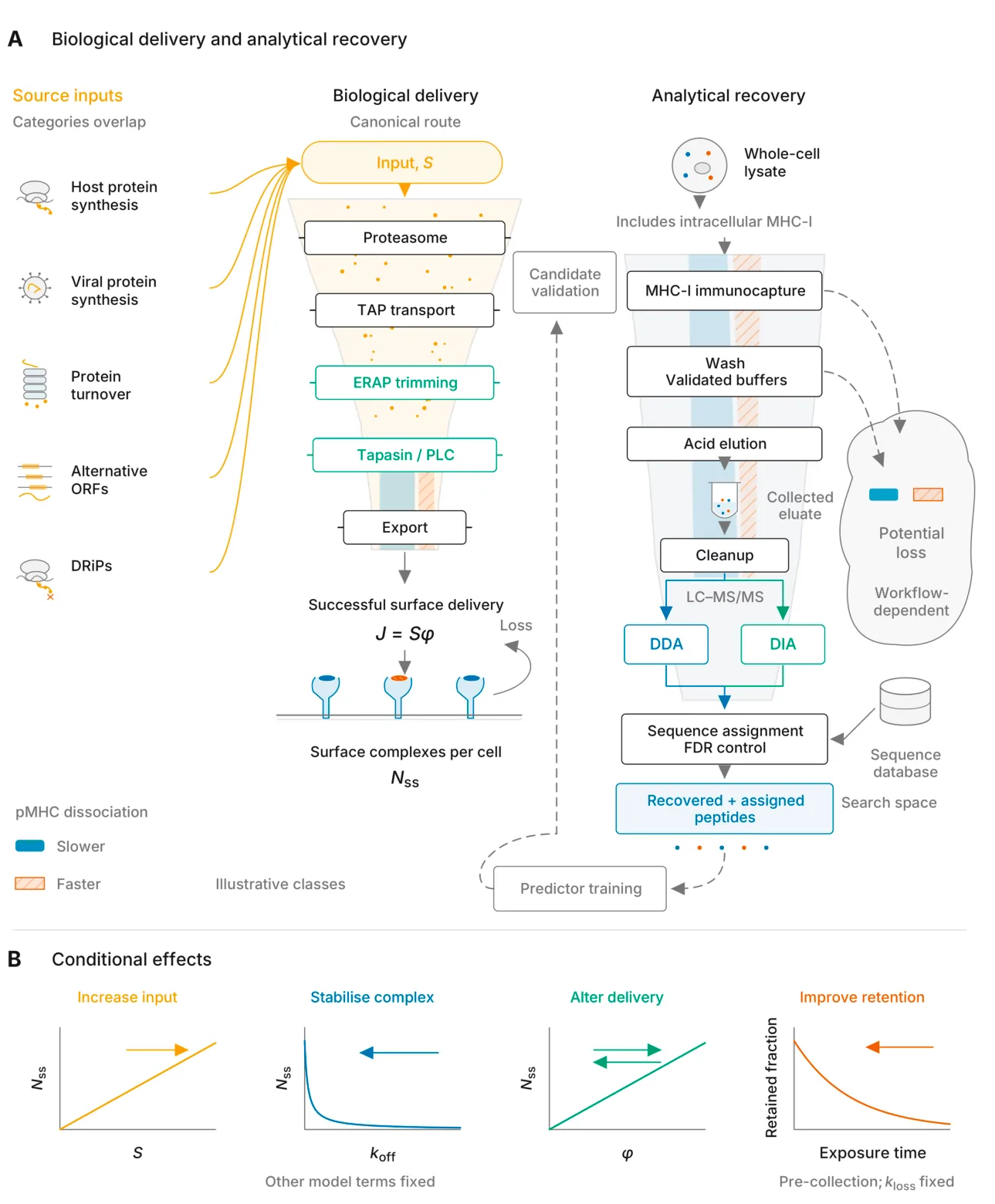
Sources of peptide loss during antigen presentation and immunopeptidomics. (**A**) Overlapping source categories contribute candidate peptide sequences to antigen processing. *S* denotes the input of copies of a candidate sequence within peptide-containing substrates. Proteolysis, transport, trimming, loading and export determine the fraction *φ* delivered as surface complexes, giving *J* = *Sφ*. Proteolysis and ER aminopeptidase (ERAP) trimming can generate or destroy a particular candidate, while tapasin influences peptide selection. Surface abundance also depends on complex loss. The analytical workflow begins with whole-cell lysate, which includes intracellular MHC-I. Recovery can be affected by dissociation during capture and washing, followed by peptide extraction, cleanup, ion sampling and sequence assignment [20,21,22,82]. Acid elution releases bound peptides for collection; it should not be interpreted as selectively discarding weak binders. DIA acts after sample preparation. Data used to train presentation predictors consequently reflect both biology and the workflow that generated them. Blue and orange marks illustrate slower and faster dissociation without assigning a measured recovery difference. (**B**) At fixed values of the other model parameters, increasing *S* or *φ* raises *N*_ss_, and reducing *k*_off_ also raises *N*_ss_. The final curve illustrates retention under an assumed constant handling-loss rate, *k*_loss_; the extent to which shorter handling improves recovery requires experimental measurement. PLC, peptide-loading complex; LC–MS/MS, liquid chromatography–tandem mass spectrometry; FDR, false discovery rate. DRiP, defective ribosomal product; ORF, open reading frame; TAP, transporter associated with antigen processing; ER, endoplasmic reticulum; MHC-I, major histocompatibility complex class I; pMHC, peptide–MHC complex; DDA, data-dependent acquisition; DIA, data-independent acquisition.

**Table 1:** Evidence relevant to peptide supply, complex stability and immune recognition.

Ligand/System	Peptide Supply Evidence	Binding or Stability Evidence	Detection Evidence	Immunological Outcome	What the Evidence Establishes
ERAAP-unedited ligands (mouse)	Peptide-specific input was not quantified	CD8^+^ T cell stimulation fell by about 80% after 2 h of brefeldin A treatment; this is a functional readout [9]	No matched capture-loss assay	Potent CD8^+^ and B cell responses; anti-pMHC mAb raised in MHC-matched mice [9]	Short-lived functional presentation can support immune recognition; supply compensation was not measured
GSW11/CT26 (mouse, H-2D^d^)	Presentation increased after ERAAP attenuation; peptide input was not measured directly [10]	Surface-stabilisation half-life of 20 min after peptide loading [23]; poor H-2D^d^ binding required a stabilised multimer reagent [24]	No matched MS recovery assay	Protective CD8-dependent immunity after ERAAP attenuation; low-avidity GSW11-specific cells were associated with anti-PD-1 efficacy [10,24,25]	Processing and regulatory T cells constrain the response; high supply compensating for rapid dissociation remains untested
NP366–374/influenza A	Presentation by multiple infected cell types; no absolute peptide-input measurement [12]	Surface-decay half-life of 223 min in a separate peptide-loading experiment [13]	Functional presentation assays	NP366-specific cells dominate the secondary response [11,12]	Presentation route contributes to the response hierarchy; the decay measurement was obtained in a separate system
PA224–233/influenza A	Presentation largely restricted to dendritic cells in the tested system [12]	Surface-decay half-life of 408 min in the same peptide-loading assay used for NP366 [13]	Functional presentation assays	PA224-specific cells contribute strongly to the primary response and less to the secondary response [11,12]	Restricted presentation can limit secondary expansion; complex lifetime alone does not establish the response hierarchy
GP276–286/LCMV	Lower relative presentation than NP396 and GP34 in the cited study [14]	Competition-assay IC_50_ of 52 nM; dissociation rate not measured [14]	Functional presentation assays	GP276-specific cells persist during chronic infection with impaired function [14]	Antigen exposure is associated with deletion or exhaustion; this does not establish supply compensation
SL9/HIV Gag	Gag abundance provides antigen substrate; SL9 input was not quantified	Escape variants can destabilise HLA-A2/SL9 complexes [26]	Binding, structural and functional measurements [26]	SL9-specific responses are prominent in chronic infection; escape can impair recognition [26,27]	Both pMHC stability and TCR recognition contribute to escape; supply compensation was not tested
Peptide stability and immunogenicity panels	Not controlled	Surface-complex decay measured at 37 °C; immunogenic peptides generally formed more stable complexes [28]	Variable	Immunogenicity was associated with complex stability [28]	Supports stability as a determinant of immunogenicity; does not isolate peptide supply
RQC-derived peptides	RQC perturbation changed presentation of a subset of peptides [29]	Not systematically measured	RQC-dependent ligands identified by MS [29]	RPS28 depletion increased sensitivity of melanoma cells to CD8^+^ T cell killing in a separate study [30]	Translation-associated processes influence peptide generation; their products are not necessarily fast dissociating
DIA-NN-only immunopeptides	Not measured independently of peptide detection	Not measured; absence from DDA does not imply low stability	Additional peptides recovered by library-based DIA-NN analysis [22]	Not tested; 85% of the DIA-NN-only 9-mers were predicted HLA binders [22]	Demonstrates additional analytical recovery; neither peptide supply nor immunogenicity was established
H-2K^b^ ligands recovered at 26 °C	Not controlled independently of temperature	Weaker predicted binding than the 37 °C peptidome (median IC_50_ 33 vs. 20 nM) [31]	Additional peptides recovered from cells cultured at 26 °C [31]	Not tested	Temperature changes the recovered repertoire; predicted affinity is not a dissociation measurement
c-Myc-derived peptides (human tumour)	High synthesis and degradation are plausible in selected models; epitope-specific flux remains unmeasured	Cannot be inferred from source-protein disorder	No detection limit or absence established here	Not tested	Candidate system for testing the model; no demonstrated fast-dissociating, supply-compensated epitope

**Notes:** The rows address different questions and are not equivalent evidence for supply compensation. Peptide supply is a rate, whereas source-protein abundance and measured pMHC abundance are amounts. Functional response decay, competition-assay IC_50_, thermal stability and peptide dissociation rate are distinct measurements. Whole-cell immunopeptidomics can include intracellular MHC-I and does not by itself establish surface copy number. MS, mass spectrometry; DDA, data-dependent acquisition; DIA, data-independent acquisition; RQC, ribosome-associated quality control; IC_50_, half-maximal inhibitory concentration; CD8, cluster of differentiation 8; ERAAP, endoplasmic reticulum aminopeptidase associated with antigen processing; Gag, group-specific antigen; HIV, human immunodeficiency virus; HLA, human leukocyte antigen; LCMV, lymphocytic choriomeningitis virus; mAb, monoclonal antibody; MHC-I, major histocompatibility complex class I; NP, nucleoprotein; PA, polymerase acidic protein; PD-1, programmed cell death protein 1; pMHC, peptide–MHC complex; TCR, T-cell receptor; RPS28, ribosomal protein S28; GP, glycoprotein; SL9, the SLYNTVATL HIV Gag epitope. DIA-NN is the analysis software used in the cited study.

**Table 2:** Evidence that peptide supply is governed by synthesis and degradation dynamics.

What It Establishes	Evidence
Rapidly degraded new proteins can supply antigenic peptides	The DRiP hypothesis explains how newly synthesised proteins can contribute rapidly to presentation [69]. Early degradation estimates were substantial [70], but their generality and interpretation remain debated [71].
New protein synthesis can be coupled to peptide supply	Translation inhibition rapidly depleted the peptide pool available to TAP in the tested system [72]. Quantitative experiments measured synthesis, degradation and presentation efficiency for model antigens [67].
Processing efficiency limits successful presentation	Cytosolic peptide destruction, TAP transport and loading restrict the fraction of degradation products presented [1]. A bulk yield is not an epitope-specific conversion factor [67].
Source abundance does not fully specify peptide input	Dynamic SILAC identified variation in the contribution of rapidly degraded products to HLA-bound peptides [66]. Measuring the source protein alone cannot resolve this contribution.
Abundance and turnover both contribute	Protein abundance and turnover were associated with HLA-I presentation [68]. Additional studies linked the repertoire to miRNA-regulated transcripts and mTOR signalling [73,74].
Dependence on degradation pathways varies between ligands	Perturbing degradation pathways revealed substantial heterogeneity in the requirements for peptide presentation [75]. The result does not imply that every ligand remains presented after pathway blockade.
Ribosome-associated processes affect the peptide repertoire	Listerin-dependent presentation identified an RQC contribution [29]. A separate ribosomal-protein perturbation study linked RPS28 depletion to altered presentation and increased melanoma-cell susceptibility to T cell killing [30].
Cellular stress can alter both antigen production and presentation	ER stress changes cellular translation and protein handling [76,77,78]. Its net effect on a particular pMHC species depends on processing and MHC-I expression, which can also be impaired in tumours [79].
Analytical recovery is distinct from biological supply	DIA identified additional immunopeptides beyond those recovered by DDA analysis [22]. Quantitative immunopeptidomics also requires correction for method-dependent measurement effects [20]. Neither measurement directly estimates peptide input.

**Notes:** Rows are grouped by the aspect of supply each addresses. DRiP, defective ribosomal product; TAP, transporter associated with antigen processing; SILAC, stable isotope labelling by amino acids in cell culture; DIA, data-independent acquisition; DDA, data-dependent acquisition; ER, endoplasmic reticulum; HLA, human leukocyte antigen; MHC-I, major histocompatibility complex class I; pMHC, peptide–MHC complex; RQC, ribosome-associated quality control; miRNA, microRNA; mTOR, mechanistic target of rapamycin; RPS28, ribosomal protein S28.

**Table 3:** Oncogenic drivers considered as peptide supply sources.

Protein	Expression in Cancer	Half-Life	Supply Implication	Condition on the Argument
c-Myc	Deregulated in many cancers [90,91,92]	Short-lived in several models, with substantial stabilisation reported in some Burkitt lymphoma lines [90,91]	High synthesis and degradation could increase peptide-containing substrate input; disorder does not predict peptide–MHC stability	Measure turnover and epitope yield in the selected line
Cyclin D1	Overexpressed in breast cancer, mantle cell lymphoma and others [93]	Approximately 20 min in the proliferating-cell model studied [94]	Continuous ubiquitin-dependent turnover regulated by GSK3β phosphorylation at Thr-286 [94]	Processing yield and the effect of altered degradation remain epitope-specific
β-catenin	Accumulation can result from impaired degradation [95,96]	Rapid turnover when the destruction pathway is active; rates depend on the model [95,96]	During accumulation after disruption of the destruction pathway, degradation flux may fall while protein abundance rises [95,96]	Compare synthesis and degradation directly when the destruction pathway is disrupted
Mutant KRAS (G12D)	Tumour-associated mutation producing characterised neoantigens [65]	No common turnover value established by the sources cited here	A supply advantage should not be assumed without measuring turnover in the system under study	For HLA-C*08:02, G12D creates a critical anchor contact with Arg156; the tested wild-type peptides did not stabilise the complex [65]

**Notes:** Half-lives are those reported in the cited studies. The final column states the condition under which the supply argument does or does not apply. None of these entries establishes matched supply compensation for an endogenous epitope. KRAS, Kirsten rat sarcoma viral oncogene homologue; HLA, human leukocyte antigen; GSK3β, glycogen synthase kinase 3 beta; MHC, major histocompatibility complex.

**Table 4:** Additional sources of MHC-I ligands and their measurement requirements.

Route	Evidence for Contribution	What Sets Supply	What Is Known About pMHC Stability	Requirements for Detection
Cryptic peptides from non-canonical ORFs	Non-canonical ligands identified by proteogenomic studies; estimated contributions depend on tissue and search strategy [82,103,104]	Expression and translation of the relevant alternative open reading frame	Non-canonical origin does not specify anchor quality or dissociation rate	Expanded sequence databases and validation of peptide assignments
Post-translational modification	Multiple classes of modified ligands identified in tumour datasets [105]	Substrate abundance and the activity of modifying enzymes	Modification can alter binding or TCR recognition; effects require measurement	Searches that include the relevant modifications and control false discovery
Proteasome-catalysed peptide splicing	Reported proportions vary substantially and remain disputed [106,107]	Proteasomal processing and joining of peptide fragments from particular substrates	Not systematically characterised	Expanded search spaces with stringent validation of candidate spliced sequences
First (pioneer) round of translation and NMD products	Antigen generation demonstrated in experimental reporter systems [108]	Translation and surveillance of the particular transcript	Instability of the source product does not establish instability of its pMHC	Transcript-aware sequence models and confirmation of endogenous presentation
Immunoproteasome peptides	Deletion of immunoproteasome subunits altered the dendritic-cell peptide repertoire [109]	Proteasome composition, substrate availability and inflammatory state	Different generated sequences can have different binding properties; no uniform direction is implied	Cell-state information and appropriate processing models; products can still be canonical sequences

**Notes:** The rows describe overlapping processes, and their reported contributions are not directly comparable. A source product that is rapidly degraded need not yield a peptide that dissociates rapidly from MHC-I. ORF, open reading frame; NMD, nonsense-mediated mRNA decay; mRNA, messenger RNA; MHC-I, major histocompatibility complex class I; pMHC, peptide–MHC complex; TCR, T-cell receptor.

**Table 5:** Predictions, experimental tests and interpretations of negative results.

Prediction	Experimental Test	Result Supporting the Prediction	Result Limiting or Challenging the Prediction
1. Increased input can compensate for faster surface loss when delivery efficiency is maintained	Inducible antigen expression and characterised variants; independent measurements of input, delivery, dissociation and total loss; calibrated surface counts and T cell assays	At comparable delivery efficiency, the input required for equal surface counts scales with total loss rate; the maintained complexes remain recognisable	The prespecified increase fails to restore presentation or recognition. Test whether measured delivery or trafficking changes explain the discrepancy
2. Some short-lived ligands are lost during sample preparation	Matched standard and shorter capture/wash workflows analysed by the same DIA method; test crosslinking separately with appropriate controls	Additional validated ligands have independently measured short complex lifetimes and handling-dependent recovery	No improved recovery within the sensitivity and lifetime range tested; this limits the proposed preparation loss in that system
3. Stabilised reagents can detect T cells missed by unstable conventional multimers	Compare conventional and validated single-chain pMHC multimers, followed by recognition of cells presenting the native antigen; GSW11 provides an established reagent example [24,25]	Additional staining identifies cells that recognise naturally processed antigen	Staining lacks native-antigen recognition, or stabilisation adds no sensitivity in the tested repertoire
4. Source synthesis and degradation improve prediction of peptide appearance	Pulsed-SILAC measurements of source proteins and ligands in selected oncoprotein models, with independent changes in synthesis and degradation [66]	Turnover measurements improve prediction beyond source abundance alone	No additional predictive value after controlling abundance and processing in the tested models
5. Altering tapasin changes which peptides are displayed and their stability distribution	Matched tapasin-sufficient, deficient and rescued cells; quantify total HLA, individual ligand abundance and measured stability	Allotype-dependent changes in repertoire composition and stability consistent with altered editing [100]	No shift in the prespecified peptide subset or stability distribution after accounting for total HLA expression
6. ERAP inhibition increases presentation of selected peptides normally destroyed by trimming	ERAP1/ERAAP perturbation with precursor processing assays, quantitative presentation and T cell recognition in defined MHC backgrounds [9,10]	Predicted trimming-sensitive ligands increase and support recognition of the perturbed cells	The selected ligands do not increase, or increased presentation does not produce the predicted recognition
7. Increased input can offset a temperature-dependent increase in loss for selected complexes	Compare 37 °C with 39–40 °C while controlling antigen induction; independently measure processing, delivery and surface loss at each temperature	For complexes with increased loss, measured additional delivery restores surface abundance within the tested range	The required delivery cannot be achieved or fails to restore the predicted count; changes in other measured processes define the limitation

**Notes:** The first experiment tests the proposed quantitative compensation under measured conditions. The remaining experiments address associated processing mechanisms or measurement limitations; some extend effects already demonstrated in particular systems. They do not independently validate the central hypothesis. Prespecify the parameter range, effect size and assay sensitivity. Negative results restrict the claim for the system tested and must not be dismissed by changing unmeasured parameters after analysis. DIA, data-independent acquisition; HLA, human leukocyte antigen; ERAP, endoplasmic reticulum aminopeptidase; ERAP1, endoplasmic reticulum aminopeptidase 1; ERAAP, endoplasmic reticulum aminopeptidase associated with antigen processing; MHC, major histocompatibility complex; pMHC, peptide–MHC complex; SILAC, stable isotope labelling by amino acids in cell culture.

**Table 6:** Prediction endpoints and measurements relevant to supply and stability.

Tool or Data Source	What It Estimates	Relation to the Model	Limitation for This Framework
NetMHCpan-4.1 [54]	Binding affinity and eluted-ligand likelihood	Binding and presentation propensity; no direct kinetic estimate	Does not directly estimate peptide input, dissociation rate or absolute surface copy number
MHCflurry 2.0 [55]	Binding and sequence-based antigen processing combined into a presentation score	Binding and processing features relevant to successful delivery	Does not directly measure protein turnover or peptide-specific input in physical units
NetMHCstab and NetMHCstabpan [34,56]	pMHC complex half-life	Predicted kinetic stability	Provides a stability estimate that requires validation for the peptide and allotype; no direct input measurement
MixMHCpred2.2 and PRIME2.0 [116,117,118]	MixMHCpred predicts presentation; PRIME adds sequence features associated with TCR recognition	Presentation and immunogenicity propensity, not direct dissociation kinetics	Scores do not supply an absolute peptide-input rate or surface count
Ribosome profiling and protein-turnover measurements [66,68,82]	Translation and turnover properties of source products	Upstream information relevant to peptide supply	Source measurements must resolve input to antigen processing; an epitope-specific yield is then needed to estimate surface delivery

**Notes:** The tools predict different endpoints and should not be treated as interchangeable measures of stability or immunogenicity. Versions listed are those described in the cited publications. Mechanistic competition models already combine supply and binding parameters [13,53]; the remaining need is independent peptide-specific measurement and validation. MHC, major histocompatibility complex; pMHC, peptide–MHC complex; TCR, T-cell receptor.

## Data Availability

This review reports no new experimental data. The model equations and parameter values underlying the illustrative figures are included in the article and figure captions.

## Abbreviations

ABCB5, ATP-binding cassette subfamily B member 5; APC, antigen-presenting cell; CD4, cluster of differentiation 4; CD8, cluster of differentiation 8; CD94, cluster of differentiation 94; CRUK, Cancer Research UK; CTL, cytotoxic T lymphocyte; DC, dendritic cell; DDA, data-dependent acquisition; DIA, data-independent acquisition; DRiP, defective ribosomal product; ER, endoplasmic reticulum; ERAAP, ER aminopeptidase associated with antigen processing; ERAP, endoplasmic reticulum aminopeptidase; ERAP1, ER aminopeptidase 1; Fab, antigen-binding fragment; FDHS, fast-dissociating high-supply; FDR, false discovery rate; Gag, group-specific antigen; GP, glycoprotein; GSK3β, glycogen synthase kinase 3 beta; HIV, human immunodeficiency virus; HLA, human leukocyte antigen; IC_50_, half-maximal inhibitory concentration; IDO1, indoleamine 2,3-dioxygenase 1; IFN-γ, interferon-γ; IP, immunoprecipitation; *J*, delivery rate of new surface pMHC complexes; *K*_D_, equilibrium dissociation constant; *k*_loss_, rate of complex loss during sample handling; *k*_other_, rate of surface complex loss by routes other than peptide dissociation; KIR, killer-cell immunoglobulin-like receptor; *k*_off_, peptide dissociation rate constant; *k*_on_, peptide association rate constant; KRAS, Kirsten rat sarcoma viral oncogene homologue; LCMV, lymphocytic choriomeningitis virus; LC–MS/MS, liquid chromatography–tandem mass spectrometry; mAb, monoclonal antibody; MHC-I, major histocompatibility complex class I; miRNA, microRNA; mRNA, messenger RNA; MS, mass spectrometry; mTOR, mechanistic target of rapamycin; NIH, National Institutes of Health; NK, natural killer; NKG2A, natural killer group 2 member A; NMD, nonsense-mediated mRNA decay; NP, nucleoprotein; *N*_ss_, steady-state number of surface complexes; ORF, open reading frame; OVA, ovalbumin-derived peptide; PA, polymerase acidic protein; PD-1, programmed cell death protein 1; PLC, peptide-loading complex; pMHC, peptide–MHC complex; PTM, post-translational modification; RQC, ribosome-associated quality control; RPS28, ribosomal protein S28; *S*, input rate of copies of the candidate sequence in peptide-containing substrates; SILAC, stable isotope labelling by amino acids in cell culture; SIV, simian immunodeficiency virus; SIY, SIYRYYGL model peptide; SL9, SLYNTVATL HIV Gag epitope; *t*_1/2_, half-life; TAP, transporter associated with antigen processing; TAPBPR, TAP-binding protein-related; TCR, T-cell receptor; *φ*, fraction of input copies delivered as surface complexes.
